# Scion genotypes exert long distance control over rootstock transcriptome responses to low phosphate in grafted grapevine

**DOI:** 10.1186/s12870-020-02578-y

**Published:** 2020-08-03

**Authors:** Antoine T. Gautier, Noé Cochetel, Isabelle Merlin, Cyril Hevin, Virginie Lauvergeat, Philippe Vivin, Alain Mollier, Nathalie Ollat, Sarah J. Cookson

**Affiliations:** 1 EGFV, Bordeaux Sciences Agro, INRAE, Univ. Bordeaux, ISVV, 33882 Villenave d’Ornon, France; 2 Crop Production and Biostimulation Laboratory, Université Libre de Bruxelles, Campus Plaine, B-1050 Brussels, Belgium; 3 Department of Biochemistry and Molecular Biology, University of Nevada, Reno, NV 89557 USA; 4 ISPA, Bordeaux Sciences Agro, INRAE, 33140 Villenave d’Ornon, France

**Keywords:** Grafting, Grapevine, Gene expression analysis, Mineral nutrition, Phosphorus, Rootstock, Scion, Viticulture, *Vitis spp*

## Abstract

**Background:**

Grafting is widely used in horticulture and rootstocks are known to modify scion growth and adaptation to soil conditions. However, the role of scion genotype in regulating rootstock development and functioning has remained largely unexplored. In this study, reciprocal grafts of two grapevine genotypes were produced as well as the corresponding homo-graft controls. These plants were subjected to a low phosphate (LP) treatment and transcriptome profiling by RNA sequencing was done on root samples collected 27 h after the onset of the LP treatment.

**Results:**

A set of transcripts responsive to the LP treatment in all scion/rootstock combinations was identified. Gene expression patterns associated with genetic variation in response to LP were identified by comparing the response of the two homo-grafts. In addition, the scion was shown to modify root transcriptome responses to LP in a rootstock dependent manner. A weighted gene co-expression network analysis identified modules of correlated genes; the analysis of the association of these modules with the phosphate treatment, and the scion and rootstock genotype identified potential hub genes.

**Conclusions:**

This study provides insights into the response of grafted grapevine to phosphate supply and identifies potential shoot-to-root signals that could vary between different grapevine genotypes.

## Background

Phosphorus (P) is an essential macronutrient for plant development involved in numerous metabolic and signalling pathways [1]. Plants are able to take up P from the soil only under its free inorganic form, orthophosphate (Pi), although many forms of P unavailable to plant uptake are present in the soil [2]. Plants have evolved several mechanisms to maximize the acquisition of Pi when resources are limiting; mechanisms ranging from modifications of plant morphology to altering gene expression. For example, many plants are able to increase the Pi foraging capacity of the root system by allocating relatively more carbon to root than shoot growth, and consequently altering shoot/root biomass ratio, and by increasing root branching and production of fine roots [3]. Plants can also increase the bioavailability of P in the soil by exuding protons, organic acids, acid phosphatases and ribonucleases to release Pi from inaccessible sources [2]. These changes are accompanied by metabolic changes in both shoots and roots, in general, the concentration of sugars, organic acids, nitrogenous compounds and secondary metabolites increase, whereas the concentration of phosphorylated metabolites decreases [4–6]. In addition, internal Pi use can be economized by the replacement of phospholipids of cellular membranes by sulfolipids and galactolipids [7].

In response to limited Pi supply, a number of genes involved in sensing, signalling and responses to Pi starvation have been identified and are called Pi-starvation-inducible (PSI) genes [8]. Lan et al. (2015) performed a meta-analysis of the response of Arabidopsis roots to P supply and identified a core of one hundred PSI genes induced hours or days after the onset of Pi starvation, independently of growth condition, experimental design and method of transcriptome analysis [8]. This list of PSI genes included genes related to lipid metabolism and galactolipid biosynthesis, transcription factors (TFs) containing SYG/PHO81/XPR1 (SPX) domains, Pi transporters (such as those from the *PHOSPHATE TRANSPORTER1*/*PHT1* family), protein kinases and intracellular secreted purple acid phosphatases [8]. The transcriptome response of perennial plants to Pi availability has received little attention despite both short and long term transcriptional responses being widely studied in annuals such as Arabidopsis, maize, rice, white lupin, tomato, bean and wild mustard [9]. To date, the only studies done on perennial crops (*Poncirus trifoliate*, *Pinus massoniana* and poplar) have been restricted to the study of only one genotype and its adaptation to a long-term (weeks to months) limiting Pi treatments [10–12]).

Comparing the responses to limited Pi supply of different genotypes can provide evidence of genetic variation of adaptive strategies and identify targets for crop improvement [13–15]. In grapevine (*Vitis spp.*), genotypes differ in their growth responses to limited Pi availability [16, 17], and in Pi acquisition and utilization efficiencies [17, 18]. Approximately one third of total plant P is exported from vineyards annually (in the berries harvested and winter pruning [19]), which suggests that grapevine is an important crop to understand the molecular basis of the genetic variation of adaptive responses to Pi supply. Furthermore, in plants amenable to grafting such as grapevine, the responses of different scion/rootstock combinations to limited Pi supply can elucidate shoot and root-specific responses to Pi supply and provide insights into the shoot and/or root signals involved. Studies using grafting on model species such as Arabidopsis have shown that both root and shoot play roles in regulating plant responses to Pi supply; potential long-distance signals include ions, metabolites, proteins, mRNAs, small RNAs and hormones [9, 20]. Some root-to-shoot signals have been identified such as Pi itself (and potentially inositol polyphosphatases and pyrophosphatases) and hormones such as strigolactones and cytokinins [9, 20]. Shoot-to-root signals include sugars, microRNAs and a calcium related signal [9, 20]. Grafting experiments have shown that under limited Pi microRNA 399 can move from the shoot to the root to suppress *PHOSPHATE2*/*PHO2* expression [21, 22]. PHOSPHATE2 mediates the protein degradation of phosphate transporters and controls shoot Pi homeostasis [23, 24]. Similarly, grafting experiments have shown that two vacuolar calcium transporters, CAX1 and CAX3, trigger a shoot-to-root signal that increases Pi uptake, but the exact mechanism remains unknown [25].

The molecular mechanisms of adaptation to low Pi supply have not yet been studied in grafted plants; here we investigate the roles of the scion and the rootstock in regulating root transcriptomic responses to low Pi supply in grafted grapevine. Firstly, we aimed to determine whether a core set of low Pi responsive transcripts could be identified across all scion/rootstock combinations studied. Secondly, we investigated whether there was genetic variation in the root transcriptomic response to low Pi supply. Thirdly, we asked whether the scion genotype could modify rootstock gene expression and its response to limited Pi supply. A weighted gene co-expression network analysis (WGCNA) allowed the identification of co-expression gene modules strongly correlated to P supply, and the rootstock and scion genotype, and identified potential hub genes.

## Results

**Fig. 1:**
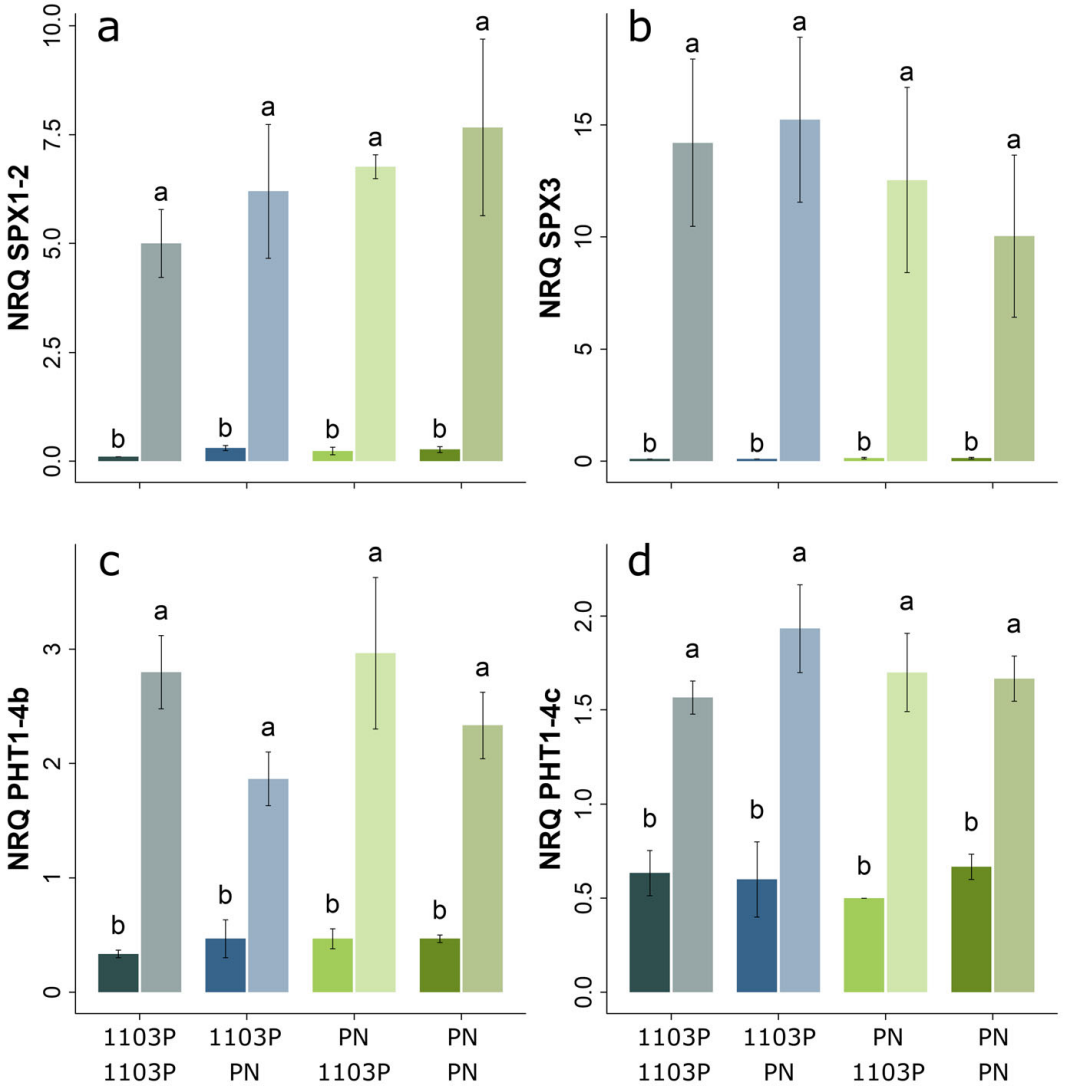
Expression of selected phosphate starvation induced genes in the root tips of grafted grapevine after 28 d of either high or low phosphate treatment (dark or light bars respectively). Gene expression was quantified by RT-qPCR and expressed as normalized relative quantities (NRQs) in reciprocal scion/rootstock combinations of *Vitis vinifera* cv. Pinot noir (PN) and *V. rupestris* x *V. berlandieri* cv. 1103 Paulsen (1103P). Means, standard deviations and results of a two-way analysis of variance (*p* < 0.05, with Tukey’s Honest Significant Difference test) are shown

Four scion/rootstock combinations of grapevine (1103P/1103P, 1103P/PN, PN/1103P and PN/PN) were grown in aerated hydroponic culture on HP, and then half of them were transferred to LP while the remaining half continued to receive HP. After 27 h treatment, RNA-seq was used to compare the short-term transcriptome response to LP in the different scion/rootstock combinations (described in detail below). And after 28 d of treatment, RT-qPCR was used to quantify the expression of four PSI genes (two SPX domain containing genes and two *PHT1* transporters) in the roots tips of plants grown on LP and HP; the expression of these genes was strongly up-regulated by LP in all scion/rootstock combinations (Fig. 1).

### A set of genes was up- or down-regulated in response to LP in all scion/rootstock combinations

**Fig. 2:**
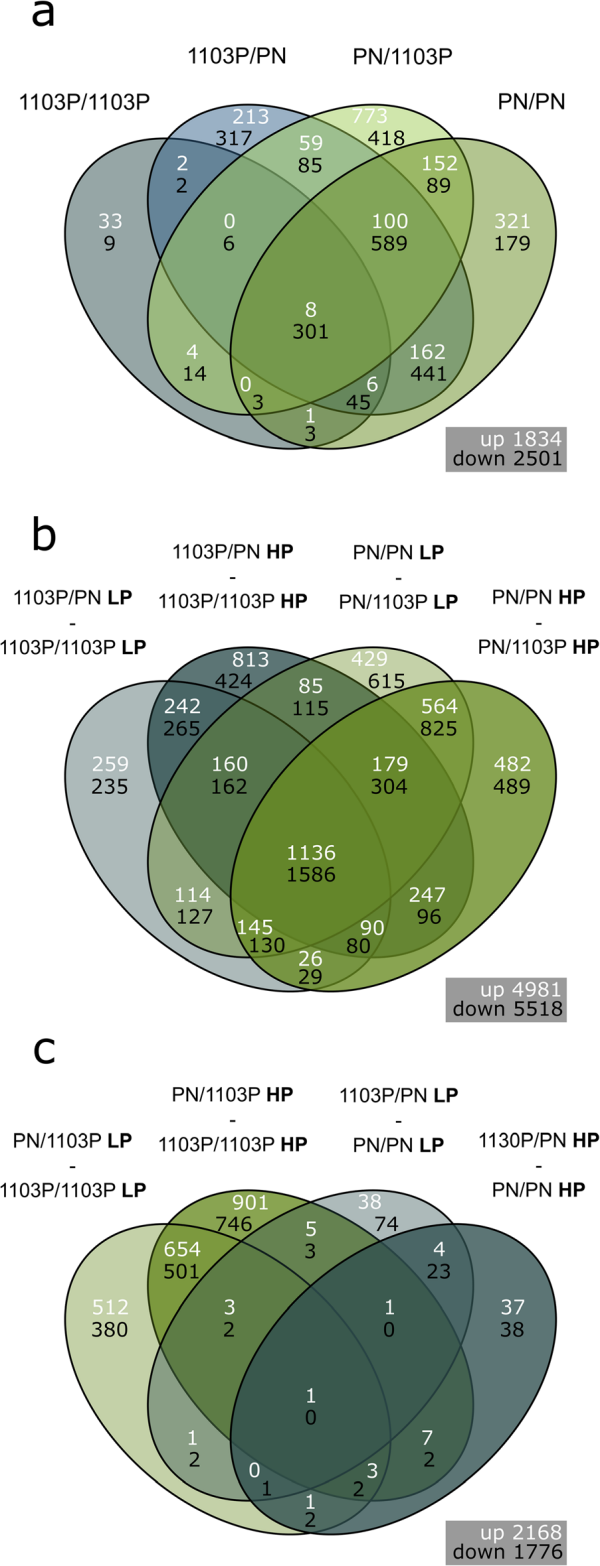
Venn diagram comparison of the number of shared and unique differentially expressed genes (log fold change > 1, false discovery rate adjusted *p*-value < 0.01) that a.) responded to 27 h low phosphate treatment in different scion/rootstock combinations of grafted grapevine, b.) were differentially expressed between the different the rootstock genotypes studied, and c.) responded to grafting with a non-self-scion. Root gene expression was studied in reciprocal scion/rootstock combinations of *Vitis vinifera* cv. Pinot noir (PN) and *V. rupestris* x *V. berlandieri* cv. 1103 Paulsen (1103P)

To characterize short-term gene expression changes in response to LP, RNA-Seq was used to quantify mRNA abundance in the root tips 27 h after transfer to LP in all scion/rootstock combinations. In comparison to the HP, the LP treatment resulted in the up- and down-regulation of the expression of 1834 and 2501 genes respectively across all scion/rootstock combinations (Fig. 2a).

A set of 301 differentially expressed (DE) genes down-regulated in response to LP was identified across all scion/rootstock combinations (Additional File 1), which was enriched in the MapMan BINs WRKY and ETHYLENE RESPONSE FACTOR/ERF TFs, members of the TYROSINE KINASE-LIKE (TKL) superfamily, PHENYLALANINE AMMONIA-LYASES/PALs and naringenin-chalcone synthases (particularly STILBENE SYNTHASES/STSs, Table 1). The WRKY TFs in this list included *WRKY6* (*Vitvi10g00063*), *WRKY18* (*Vitvi04g00510*), *WRKY33* (*Vitvi06g00741* and *Vitvi08g00793*), *WRKY40* (*Vitvi04g00511* and *Vitvi09g01122*), *WRKY51* (*Vitvi04g00760* and *Vitvi04g01985*), *WRKY53* (*Vitvi15g01003* and *Vitvi16g01213*), and *WRKY75* (*Vitvi01g01680*). There were 16 ERF TFs in this list as well as other genes associated with ethylene signalling such as *1-AMINOCYCLOPROPANE-1-CARBOXYLATE OXIDASE 1*/*ETHYLENE-FORMING ENZYME* (*Vitvi12g00445*) and the ethylene responsive gene, *INFLORESCENCE DEFICIENT IN ABSCISSION* (*Vitvi01g01924*). In addition, this list included genes related to secondary metabolism, such as *PREPHENATE DEHYDRATASE* (*Vitvi06g01946*), which is involved in the biosynthesis of aromatic amino acids such as phenylalanine, and two *CINNAMATE-4-HYDROXYLASES*/*C4Hs* (*Vitvi11g00924* and *Vitvi11g01045*) and two *4-COUMAROYL COA-LIGASES*/*4CLs* (*Vitvi14g01757* and *Vitvi02g00717*) which are involved in phenylpropanoid metabolism.

**Table 1:** MapMan BINs enriched in the 301 genes down-regulated in the roots in response to 27 h of a low phosphate treatment in the four scion/rootstock combinations of grafted grapevine studied (PN/PN, PN/1103P, 1103P/PN and 1103P/1103P). PN: *Vitis vinifera* cv. Pinot noir; 1103P: *V. rupestris* x *V. berlandieri* cv. 1103 Paulsen

MapMan BIN	MapMan BIN name	Enrichment	Adjusted *p*-values
15.7.22	RNA biosynthesis.transcriptional activation. WRKY transcription factor	25.7	0.00
15.7.7.1	RNA biosynthesis.transcriptional activation. AP2/ERF superfamily. ERF-type transcription factor	28.5	0.00
18.8.1.19	Protein modification.phosphorylation. TKL kinase superfamily. L-lectin kinase	13.6	0.02
18.8.1.23	Protein modification.phosphorylation. TKL kinase superfamily. RKF3 kinase	92.2	0.01
18.8.1.45	Protein modification.phosphorylation. TKL kinase superfamily. RLCK-Os kinase	59.5	0.00
9.2.1.1	Secondary metabolism.phenolics.p-coumaroyl-CoA synthesis.phenylalanine ammonia lyase (PAL)	65.6	0.00
9.2.2.1	Secondary metabolism.phenolics.flavonoid synthesis and modification.chalcone synthase	41.9	0.00

Only 8 DE genes were up-regulated in response to LP in all the scion/rootstock combinations, these genes were: an ABC transporter (*Vitvi14g01865*), a receptor kinase (*FERONIA, Vitvi14g01365*), a disease resistance protein (*Vitvi18g01755*), two transferases (*Vitvi12g02284* and *Vitvi07g03041*), *ISOFLAVONE 2′-HYDROXYLASE*/*CYP81D8* (*Vitvi07g01651*) and a putative transcriptional regulator (the transposase-like protein *Vitvi13g02203*) (Additional File 1). In summary, the DE genes down-regulated in response to LP were well conserved between the scion/rootstock genotypes studied, but the DE genes up-regulated in response to LP were largely scion/rootstock combination specific.

### Genes differentially regulated between the two homo-grafts in response to LP

Many genes were DE between the two rootstock genotypes under both HP and LP (Fig. 2b, Additional File 2). Under the control HP conditions, the genes DE between the two rootstock genotypes were enriched in the MapMan BINs related to enzymes and nucleotide-binding domain and leucine-rich repeat-containing (NLR) effector receptors (which perceive pathogen infection to trigger immunity mechanisms). In addition, the MapMan BINs PAL and chalcone synthase were also enriched in the genes more highly expressed in PN (Additional File 3).

**Fig. 3:**
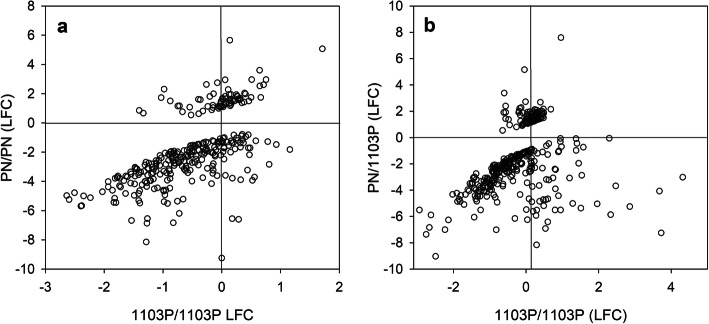
Relationship between a.) the transcriptional responses to 27 h low phosphate treatment in PN/PN and 1103P/1103P of the genes showing a significant interaction (log fold change > 1, false discovery rate adjusted *p*-value < 0.01) between homograft genotype and phosphate treatment, and b.) the transcriptional responses to 27 h low phosphate treatment in PN/1103P and 1103P/1103P of the genes showing a significant interaction between the scion genotype and the phosphate treatment. The scion/rootstock combinations studied were grafts of PN (*Vitis vinifera* cv. Pinot noir) and 1103P (*V. rupestris* x *V. berlandieri* cv. 1103 Paulsen)

The comparison of the transcriptome response to LP between the two homo-grafts (PN/PN and 1103P/1103P) clearly demonstrated that more genes were DE in PN/PN than in 1103P/1103P (Fig. 2a). To determine whether the transcriptome response to LP was different between the homo-grafts, interactions were calculated (i.e. the genes DE between (PN/PN LP versus PN/PN HP) versus (1103P/1103P LP versus 1103P/1103P HP), Additional File 4). The expression of 310 genes differently responded to LP between the two homo-grafts (Additional File 4), these 310 genes were enriched in the MapMan BINs NAC and WRKY TFs, solute transport (VACUOLAR IRON TRANSPORTER/VIT and ALUMINIUM ACTIVATED MALATE TRANSPORTER families), biotic stress, oxidoreductases (mainly orthologues of LACCASE 14, which is involved in lignin degradation) and transferases (that transfer a P containing group) (Additional File 5). The BINs from the biotic stress category were NLR effector receptors and the ERN1 transcription factor (which regulates rhizobial infection in *Lotus japonicas* [26]). To characterize the expression patterns of these 310 genes showing a significant interaction in response to LP between the homo-grafts, the log fold change of gene expression in response to LP in 1103P/1103P was plotted against PN/PN (Fig. 3a). Globally, the DE genes down-regulated in response to LP were qualitatively well-conserved between the two homo-grafts, with 67 DE genes significantly down-regulated in both PN/PN and 1103/1103P, but the fold change was higher for PN/PN. The DE genes more highly down-regulated in response to LP in PN/PN than 1103P/1103P included many TFs, kinases, biotic stress associated genes and genes related to calcium signalling. The DE genes up-regulated in response to LP were less conserved between the homo-grafts with only one gene (a methyl jasmonate esterase, *Vitvi07g02517*) significantly up-regulated in response to LP in both homo-grafts, but with a higher fold change in PN/PN. The DE genes more highly up-regulated in response to LP in PN/PN than 1103P/1103P included *WRKY19* (*Vitvi18g01743*), a sulphate transporter (*Vitvi09g00484*), the Snf1 protein kinase *KIN10* (*Vitvi08g00935*) and a large subunit of ADP-glucose pyrophosphorylase (*Vitvi07g00544*) which regulates starch synthesis as well as genes associated with jasmonate, cytokinins and abscisic acid (ABA) signalling. Only one gene (*Vitvi08g01380*) was up-regulated in response to LP in 1103P/1103P and down-regulated in PN/PN, and vice versa only one gene (*Vitvi18g02447*) was down-regulated in response to LP in 1103P/1103P and up-regulated in PN/PN. In conclusion, the transcriptome response to LP of PN/PN was more extensive than that of 1103P/1103P, but the qualitative change in gene expression is similar between the two homo-grafts, particularly for the down-regulated genes.

### Genes differentially regulated in response to grafting with a non-self-scion

Grafting with a non-self-scion triggered the differential expression of many genes; however one gene, a BURP domain-containing protein RD22-like isoform X2, *VViBURP17* (*Vitvi04g00357*) [27] was DE in response to hetero-grafting in both rootstocks and P treatments (Fig. 2c, Additional File 6). BURP domain containing proteins are involved in abiotic stress tolerance in several plant species and are generally responsive to ABA application [27].

When comparing 1103P/PN and PN/PN, only a small number of DE genes responded to grafting with a non-self-scion in both LP and HP in the PN rootstock; 6 and 24 DE genes were up and down-regulated respectively (Fig. 2c). The six transcripts that increased in abundance in the 1103P/PN relative to PN/PN included *CAROTENOID CLEAVAGE DIOXYGENASE 8* (*Vitvi04g00298*), an enzyme involved in the synthesis of strigolactones (Additional File 7). The 24 transcripts that decreased in abundance were enriched in the MapMan BINs meiotic exit regulator (involved in cell cycle), protein modification and iron uptake (Additional File 7).

When comparing PN/1103P and 1103P/1103P, a large transcriptional response to the grafting with a PN scion was detected with 661 and 505 DE genes up- and down-regulated respectively under both LP and HP (Fig. 2c, Additional File 8). The 661 DE genes that were up-regulated in PN/1103P compared to 1103P/1103P were enriched in the MapMan BINs DNA-binding with one finger, NAC, basic helix-loop-helix/bHLH and homeobox TFs, TKL kinases, subtilisin-like proteases (which are associated with biotic stress responses), proteins involved in cellulose synthesis, heat shock proteins and galactinol synthase (Additional File 8). Galactinol synthase mediates raffinose accumulation, an osmoprotectant that promotes stress tolerance [28]. The 505 DE genes that were down-regulated in PN/1103P compared to 1103P/1103P were enriched in the MapMan BINs GATA TFs, bHLH TFs, GROWTH REGULATING FACTORS, serine carboxypeptidases, hydrolases and precursors of cysteine rich peptides (Additional File 8). Cysteine rich peptides are involved in regulating a range of developmental responses in plants and can be involved in cell-to-cell communication, particularly in response to the establishment of plant-bacteria symbiosis and root development [29].

Overall, the transcriptome of the rootstock responds to grafting with a non-self-scion, but the degree of transcriptome response is scion/rootstock dependent and there does not appear to be a general non-self-scion response of the rootstock transcriptome.

### The impact of the scion on the root transcriptome response to LP

The comparison of the response to LP of PN/PN and 1103P/PN showed that the scion can have a slight impact on the transcriptome response of the rootstock to LP (Fig. 2a). However, the response to LP was quantitatively different for only five genes between PN/PN and 1103P/PN (i.e. genes DE between (1103P/PN LP versus 1103P/PN HP) versus (PN/PN LP versus PN/PN HP), Additional File 9). The four genes were more down-regulated in 1103P/PN than PN/PN were two ABCG40 transporters (*Vitvi09g00473* and *Vitvi09g00478*), a gene involved in tyrosine synthesis (*Vitvi12g00272*) and an UDP-GLYCOSYLTRANSFERASE encoding gene (*Vitvi12g01724*) assigned to the MapMan BIN ABA synthesis/degradation.

In contrast, the comparison of the response to LP of 1103P/1103P and PN/1103P showed that the PN scion dramatically increased the transcriptome response of the 1103P rootstock to LP; only 59 and 42 transcripts specifically decreased and increased in abundance in 1103P/1103P, whereas 1181 and 1084 transcripts decreased and increased in abundance in PN/1103P respectively. To determine whether the transcriptome response to LP was quantitatively different between the 1103P/1103P and PN/1103P interactions were calculated (i.e. the genes DE between (PN/1103P LP versus PN/1103P HP) versus (1103P/1103P LP versus 1103P/1103P HP), Additional File 10). Only 364 DE genes differently responded to LP, these genes were enriched in the MapMan BINs ERF TFs, NLR effector receptors and phosphoserine aminotransferases (which are involved in serine biosynthesis) (Table 2). As was seen in the comparison between the transcriptional responses to LP of the homo-grafts, there was a conservation of response in the LP down-regulated genes (Fig. 3b), with 113 DE genes significantly down-regulated in PN/1103P and 1103P/1103P, but the gene expression response of PN/1103P was of higher magnitude. However, no common DE genes were significantly up-regulated in response to LP in the genes showing an interaction (Fig. 3b); the DE of 12 genes was reduced in response to LP in PN/1103P and increased in response to LP in 1103P/1103P.

**Table 2:** MapMan BINs enriched in the 364 genes differentially expressed in the roots between the scion/rootstock combinations PN/1103P and 1103P/1103P in response to 27 h of a low phosphate treatment. PN: *Vitis vinifera* cv. Pinot noir; 1103P: *V. rupestris* x *V. berlandieri* cv. 1103 Paulsen

BIN	NAME	Enrichment	Adjusted *p*-values
15.7.7.2	RNA biosynthesis.transcriptional activation. AP2/ERF superfamily. DREB-type transcription factor	10.9	0.04
26.6.2.1	External stimuli response.biotic stress.pathogen effector. NLR effector receptor	5.4	0.01
4.1.4.1.2	Amino acid metabolism.biosynthesis.serine family.non-photorespiratory serine.phosphoserine aminotransferase	114.3	0.01

The 104 DE genes more up-regulated in PN/1103P than 1103P/1103P included a cyclin (*Vitvi15g00641*), the bHLH TF *FIZZY-RELATED 3* (*Vitvi12g02634*), an *ALUMINUM ACTIVATED MALATE TRANSPORTER* (*Vitvi13g02145*), four *DICER-LIKE3* genes, *FAR-RED IMPAIRED RESPONSE1-RELATED SEQUENCE5* (*Vitvi08g01733*) and many resistance proteins and genes involved in biotic stress responses and (Additional File 10).

The 260 DE genes more down-regulated in PN/1103P than 1103P/1103P included genes related to amino acid metabolism, development (including a VIT family protein), fermentation and glycolysis (cytosolic branch e.g. *PYRUVATE KINASE* (V*itvi10g01568*), *PHOSPHOGLYCERATE KINASE* (*Vitvi19g01690*) and *GLYCERALDEHYDE-3-PHOSPHATE DEHYDROGENASE* (*Vitvi01g01538*)) (Additional File 10). This list also included five ERF TFs, a zinc finger homeobox TF (*Vitvi12g02000*), a *NADP-DEPENDENT MALIC ENZYME* (*Vitvi04g00009*), two EXORDIUM-like 2 encoding genes, and a *TREHALOSE-6-PHOSPHATE PHOSPHATASE* (*Vitvi18g00384*) (Additional File 10).

In summary, the scion can modify rootstock transcriptome responses to LP, but its effect is rootstock dependent.

### Gene co-expression network analysis

**Fig. 4:**
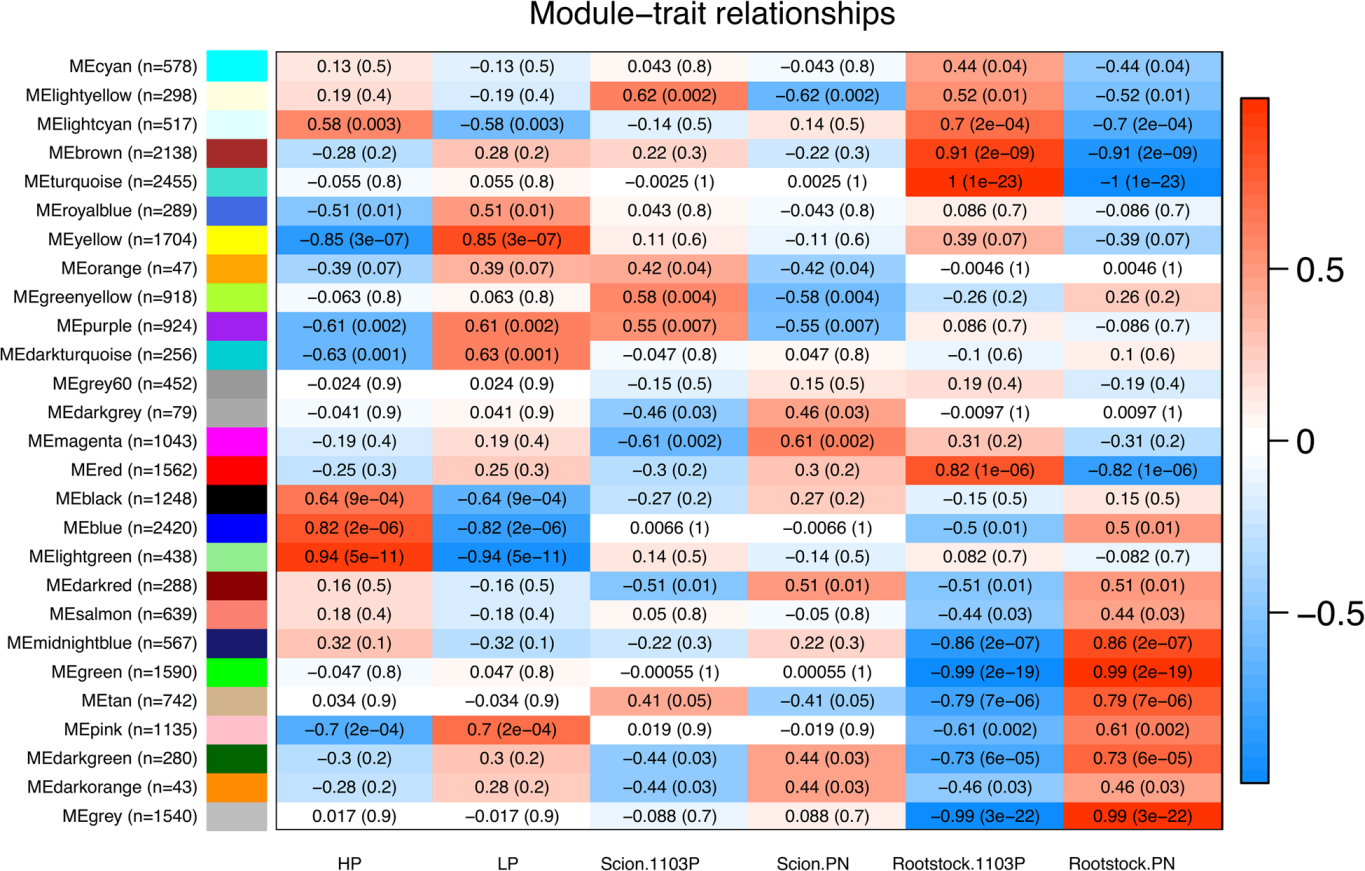
Module-trait relationships from the weighted gene co-expression network analysis. Module-phosphate treatment/scion/rootstock genotype correlation coefficients and corresponding *p*-values (in parenthesis) are given in each cell. The left panel shows the 26 modules and the number of module member genes. The colour scale on right shows module-trait correlation from −1 (blue) to 1 (red). HP: high phosphate, LP: low phosphate; PN: *Vitis vinifera* cv. Pinot noir; 1103P: *V. rupestris* x *V. berlandieri* cv. 1103 Paulsen (1103P)

A WGCNA approach was used to identify 26 modules of highly correlated genes and these modules were analysed for their association with each experimental variable (the P treatment, and scion and rootstock genotype) (Fig. 4). Genes with highly correlated expression patterns were identified based on kME values (Additional File 11) those with the highest correlation coefficients (> 0.9) could be considered as potential hub genes within the module. The module MEgrey contained the genes which could not be assigned to a co-expression module.

### Gene co-expression modules related to P supply

The gene co-expression module the most positively correlated to LP was MEyellow; which contained 1704 genes (Fig. 4). The highly correlated genes (> 0.8) were enriched in the MapMan BINs transcriptional activation, particularly C3H zing finger and FAR-RED IMPAIRED RESPONSE 1 TFs (Table 3). The most 144 highly correlated genes (> 0.9) included genes involved in RNA (particularly small RNA) processing (such as *DICER-LIKE1* and *DICER-LIKE3*), chromatin remodelling factors, two histone acetyltransferases (*Vitvi12g00328* and *Vitvi19g00124*), *DEMETER* (*Vitvi08g01515*), three genes from the MapMan BIN chromatin structure as well as other biotic and abiotic stress responsive transcripts (Additional File 12). The co-expression module most positively correlated with HP was MElightgreen, which contained 438 transcripts. The positively correlated transcripts (> 0.8) were enriched in the MapMan BINs WRKY and ERF TFs, and chalcone synthase (Table 3).

**Table 3:** MapMan BINs enriched in the most highly positively correlated (> 0.8) genes in selected modules of the weighted gene co-expression network analysis made from the genes expressed in the roots of different scion/rootstock combinations of grapevine grown under two different phosphate supplies

Module	BIN	Name	Enrichment	Adjusted *p*-value
MEyellow	15.7.16	RNA biosynthesis.transcriptional activation. C3H zinc finger transcription factor	6.1	0.02
	15.7.49	RNA biosynthesis.transcriptional activation. FAR1 transcription factor	6.6	0.00
MElightgreen	15.7.22	RNA biosynthesis.transcriptional activation. WRKY transcription factor	7.5	0.00
	15.7.7.1	RNA biosynthesis.transcriptional activation. AP2/ERF superfamily. ERF-type transcription factor	11.7	0.00
	9.2.2.1	Secondary metabolism.phenolics.flavonoid synthesis and modification.chalcone synthase	15.3	0.00
MEturquoise	26.6.2.1	External stimuli response.biotic stress.pathogen effector. NLR effector receptor	3.5	0.00
	50.1.13	Enzyme classification.EC_1 oxidoreductases.EC_1.14 oxidoreductase acting on paired donor with incorporation or reduction of molecular oxygen	2.0	0.00
	50.2.1	Enzyme classification.EC_2 transferases.EC_2.1 transferase transferring one-carbon group	2.6	0.00
	50.2.4	Enzyme classification.EC_2 transferases.EC_2.4 glycosyltransferase	2.1	0.00
	50.3.2	Enzyme classification.EC_3 hydrolases.EC_3.2 glycosylase	2.0	0.03
MEgreen	16.9.1.2.1	RNA processing.messenger ribonucleoprotein particle (mRNP).mRNP export. TREX-2 mRNP trafficking complex. GANP/SAC3 scaffold component	9.1	0.00
	26.6.2.1	External stimuli response.biotic stress.pathogen effector. NLR effector receptor	2.4	0.01
	50.1.13	Enzyme classification.EC_1 oxidoreductases.EC_1.14 oxidoreductase acting on paired donor with incorporation or reduction of molecular oxygen	1.9	0.00
	9.1.3.1	Secondary metabolism.terpenoids.terpenoid synthesis.mono−/sesquiterpene−/diterpene synthase	3.9	0.04
MEgreenyellow	11.10.2.1.1	Phytohormones.signalling peptides. CRP (cysteine-rich-peptide) category. GASA/GAST family. GASA precursor polypeptide	16.0	0.01
	15.7.35.1	RNA biosynthesis.transcriptional activation. GRF-GIF transcriptional complex. GRF transcription factor component	35.5	0.00
	19.5.2.2	Protein degradation.peptidase families.serine-type peptidase activities.serine carboxypeptidase	8.2	0.00
	21.3.1.1.3	Cell wall.pectin.homogalacturonan.synthesis. CGR-type methyltransferase	33.0	0.01
	21.4.1.1.2.2	Cell wall.cell wall proteins.hydroxyproline-rich glycoproteins.arabinogalactan proteins (AGPs).glycoproteins.fasciclin-type arabinogalactan protein	11.7	0.05
MEmagenta	11.9.2.3	Phytohormones.strigolactone.perception and signal transduction. SMXL signal transducer	20.2	0.04
	15.7.1.5	RNA biosynthesis.transcriptional activation. C2C2 superfamily. DOF transcription factor	10.6	0.00
	15.7.17	RNA biosynthesis.transcriptional activation. NAC transcription factor	4.6	0.05
	15.7.3.5	RNA biosynthesis.transcriptional activation. HB (Homeobox) superfamily. BEL transcription factor	12.4	0.03
	15.7.33	RNA biosynthesis.transcriptional activation.bHLH transcription factor	3.9	0.02
	18.8.1.11	Protein modification.phosphorylation. TKL kinase superfamily. LRR-XI kinase	9.6	0.02

### Gene co-expression modules related to the transcriptome differences between the rootstock genotypes

The co-expression module that was most highly positively correlated with the rootstock 1103P is MEturquoise; this module contained 2455 genes (Fig. 4). The highly correlated genes (> 0.8) were enriched in the MapMan BINS associated with NLR effector receptors and various enzymes (Table 3). The co-expression module that was most highly positively correlated with the rootstock PN is MEgreen, containing 1590 genes and the highly correlated genes (> 0.8) were enriched in the MapMan BINs involved with RNA processing, NLR effector receptors and various enzymes (Table 3).

### Gene co-expression modules related to the effect of the scion genotype on the root transcriptome

Fewer gene co-expression modules were significantly correlated with the scion genotype and the correlation coefficients were much lower than for the P treatment or rootstock genotype (Fig. 4). The module MElightyellow was the most highly correlated with the 1103P scion (298 transcripts) but was also significantly correlated with the rootstock genotype. Whereas the module MEgreenyellow was only significantly correlated with the scion genotype so the genes are more likely to be scion response specific. MEgreenyellow was enriched in the MapMan BINs signalling peptides (cysteine rich peptides), transcriptional activation, protein degradation (serine carboxypeptidases) and cell wall (Table 3). The 121 most highly positively correlated transcripts (> 0.9) included five *GROWTH REGULATING FACTORS*, which are potentially targeted by the microRNA 396 [30] (Additional File 12). The module MEmagenta was the co-expression module most highly correlated with the PN scion (736 transcripts), the MapMan BINs strigolactone signalling, C2C2, NAC, bHLH and BEL TFs, and some TKL receptor kinases were enriched in this module (Table 3).

## Discussion

The availability of a reference genome for grapevine is a great resource for RNAseq analysis, but our knowledge is limited to the predicted transcriptome of this reference genome (i.e. PN40024). The literature suggests that genomic variations are significant even at the clonal level [31]. Sequencing the clone of 1103P that was used in this study would help correct for transcript quantification of highly duplicated genes/gene families, but was beyond the scope of this project. However, despite differences between genomes of the same genus, pan-genome studies have showed that there is a high proportion of orthologous genes shared between related species [32, 33].

### Many PSI genes from Arabidopsis were not induced in response to 27 h of LP in grapevine

In this study on grapevine, we identified a set of 301 and eight DE genes that were down and up-regulated respectively in all scion/rootstock combinations. Surprisingly, there were few of the PSI genes of Arabidopsis [8] in this dataset, this is partly because the grapevine orthologues have not been identified (e.g. *INDUCED BY PHOSPHATE STARVATION1IPS1* and *IPS2*) or they were expressed at very low levels (e.g. *MITOGEN-ACTIVATED PROTEIN KINASE KINASE KINASE 19*). However, some orthologues of Arabidopsis PSI genes were not DE, e.g. *PHOSPHATE STARVATION RESPONSE1-LIKE1* (*Vitvi07g00666* and *Vitvi14g00736*) and the two orthologues of *PHO2* (*Vitvi00g02237* and *Vitvi10g01764*, which were not significantly DE, but were slightly up-regulated under LP particularly in PN/PN and PN/1103P). Orthologues of *WRKY6*, *WRKY18*, *WRKY40* and *WRKY75* TFs were present in the set of 301 genes that were down-regulated in response to LP in all scion/rootstock combinations, although these genes are PSI genes in Arabidopsis and have roles in regulating Pi starvation responses [34–36]. However, after 28 d of LP, the expression of orthologues of four Arabidopsis PSI genes was up-regulated relative to HP suggesting that the response to LP at 27 h represents the early stress-related changes in gene expression.

### A set of 309 genes that were DE in response to LP in all scion/rootstock genotypes was identified

The down-regulation of gene expression in response to LP was well-conserved between the four scion/rootstock combinations studied (301 genes), whereas the expression of only eight genes was up-regulated in response to LP across all scion/rootstock combinations. Messenger RNA stability plays an important role in the regulation of gene expression. Short-lived transcripts (such as TFs and kinases) are often associated with regulatory processes (whereas longer-lived transcripts tend to be associated with protein synthesis and energy balance) [37, 38]. It has previously been shown that transcript degradation makes a large contribution to the rapid response to sucrose in Arabidopsis seedlings [39] and is involved in a wide range of abiotic stress responses (as reviewed by [40]. The set of 301 genes down-regulated in response to LP in all scion/rootstock combinations was enriched in TFs and kinases suggesting that their down-regulation could be related to their rapid turnover. It seems that conservation of genes down-regulated in response to LP between the different scion/rootstock combinations is important and this could be because rapid transcript turnover has the potential to ensure quick responses to changing soil nutrient conditions.

Many genes involved in the pathway of stilbene synthesis were present in the set of 301 DE genes down-regulated in response to LP in all scion/rootstock combinations: for example one *PREPHENATE DEHYDRATASE*, nine *PALs*, two *C4Hs*, two *4CLs* and 13 *STSs*. Many metabolites are known to be differentially accumulated in plants in response to LP, a general response to increase the concentration of secondary metabolites (particularly the concentration of anthocyanins in leaves in response to LP supply [41]), but little is known about whether stilbene synthesis is altered in response to LP because only few plants produce stilbenes. To date, there has only been one study of a plant that produces stilbenes, this study was done on pine trees, after a longer term low Pi treatment and did not highlight the differential expression of genes related to secondary metabolism [11]. Stilbenes are known to have roles in plant defence responses [42], so this down-regulation of expression of genes related to stilbene synthesis 27 h after transfer to a LP treatment may be an early response to redirect metabolism from defence responses to adapting to limited P. Many ethylene related genes were also present in the set of 301 DE genes that were down-regulated in response to LP in all scion/rootstock combinations; these genes are known to have a wide range of roles in both biotic and abiotic stress responses, including regulating root growth responses to P supply (as reviewed by [9]). In the WGCNA analysis, the module most strongly positively correlated with HP was MElightgreen; the highly correlated genes in this module were enriched in the MapMan BINs WRKY and ERF TFs and chalcone (stilbene) synthases. Both WRKY TFs and ethylene have been shown to regulate the expression of *STSs* in grapevine [43] suggesting that these TFs could be hub genes regulating metabolomic responses to LP. In addition, *VviMYB15* (*Vitvi05g01733*) and *VViMYB14* (*Vitvi07g00598*), positive regulators of *STSs* [44], were down-regulated in most scion/rootstock combinations. The down-regulation of expression of *VviMYB14* and many *STSs* has also been observed in grapevine in response to iron deficiency, these changes were accompanied by an increase in the expression of genes involved in flavonoid synthesis [45]. Similarly, one of the eight DE genes that were up-regulated in all scion/rootstock combinations in response to LP was *ISOFLAVONE 2′-HYDROXYLASE/CYP81D8* (*Vitvi07g01651*). This suggests that at the transcriptional level the down-regulation of stilbene synthesis and the up-regulation of flavonoid synthesis are common responses to Pi and iron deficiency in grapevine roots.

The expression of only eight genes was up-regulated in response to LP in all scion/rootstock combinations, this suggest that there was more variation in the up- than the down-regulation of gene expression in response to LP of individual scion/rootstock combinations. Despite this variation of transcriptome response, the WGCNA analysis allowed the identification of modules positively correlated with the LP treatment. The most strongly positively correlated module with LP was MEyellow; the genes in this module were enriched in the MapMan BINs transcriptional activation including FAR-RED IMPAIRED RESPONSE1. FAR-RED IMPAIRED RESPONSE1 is known to be involved in phytochrome signalling, but it has recently been shown to regulate the *PHR1* expression (along with ethylene) in Arabidopsis [46]. This could suggest that FAR-RED IMPAIRED RESPONSE1 TFs regulate early LP transcriptome responses in grapevine. Many genes involved in chromatin modification and RNA processing such as *DICER-LIKE1* and *DICER-LIKE3* genes were present in the list of genes most positively correlated with MEyellow. In Arabidopsis, DICER-LIKE1 is involved in the formation of microRNAs, which are known to be differentially produced in response to Pi starvation [9]. DICER-LIKE3 generates siRNAs that direct DNA methylation and has been shown to be inhibited by Pi starvation [47]; the presence of *DICER-LIKE3* genes in MEyellow suggests that siRNA-directed DNA methylation may also be important in the response of grapevine to P availability. DNA methylation is known to be important in the regulation of Pi starvation responses in both annual [48] and perennial species [49]. Chromatin modifications are implicated in the regulation of PSI gene expression in Arabidopsis [50] and one transposase-like protein was present in the set LP up-regulated genes in all scion/rootstock combinations suggesting that this may also be important in grapevine.

### Do grapevine genotypes differ in their response to LP?

Many genes were DE in the roots between the two different genotypes studied under both HP and LP conditions, and the modules MEturquoise and MEgreen were associated with the rootstocks 1103P and PN respectively. The genes DE between the two rootstocks were associated with various enzymes and NLR effector receptors, similar results have been acquired in other genotype-specific transcriptome comparisons in grapevine [51, 52]. The two homo-grafts also differed in their transcriptome response to P supply; the transcriptome response of PN/PN was more extensive than that of 1103P/1103P after 27 h of LP treatment although the qualitative changes in gene expression were similar between the two genotypes. Genes related to iron, organic acid and sulphate transport, and carbon metabolism were DE between the two homo-grafts along with genes associated with hormone signalling. The large number of TFs was DE between the two homo-grafts in response to LP suggesting that the genotypes differ in the TF networks employed to regulate responses to LP.

### Does the scion genotype modify the rootstock transcriptome and its response to P supply?

There have been a number of studies in the literature of how rootstocks alter gene expression in the scion of perennial [53–57] and annual crops [58], and the responses of the scion to different environmental conditions [59, 60]. However, few studies have been done the other way round, to investigate how the scion modifies the transcriptome of the rootstock and its response to the environment. In this study we identified only one transcript, *VViBURP17*, which increased in abundance in all hetero-grafts in comparison to the corresponding homo-graft control. This suggests that there no conserved root transcriptome response to grafting with a non-self-scion and that each rootstock responds differently.

In grapevine, grafting with non-self-rootstocks triggers the differential expression of genes involved in defence and stress responses (such as genes encoding the biosynthesis of secondary metabolites and receptor kinases), chromatin modification, transcriptional regulation and hormone signalling [53, 54] in the scion. Similar genes were DE in the root in response to grafting with a non-self-scion. In addition, some of the scion-responsive transcripts and potential hub genes identified in the WGCNA suggest that the scions used in this study alter cysteine rich peptide signalling and microRNA production (particularly microRNA 396). It is also possible that some of the scion-responsive transcripts are in fact mRNAs that have moved from the scion to the rootstock, such movement is complex to understand and in tobacco/tomato grafts seems to involve both a regulated and un-regulated components [61]. We assume that these defence and stress-related scion-responsive transcripts are not related to problems of incompatibility because grapevine grafts relatively easily in in vitro culture and the treatments were applied 10 weeks after grafting.

The impact of the scion on the rootstock response to LP was dependent on the rootstock studied; only six genes were DE between 1103P/PN and PN/PN in response to LP, whereas 364 genes were DE between PN/1103P and 1103P/1103P. This is largely because few genes were DE in response to LP in the 1103P/1103P in comparison to the other scion/rootstock combinations. In general, the qualitative change in gene expression of these 364 was similar between the two scion genotypes, particularly for the genes down-regulated in response to LP. The enrichment of MapMan BINs in the 364 genes DE in response to LP between PN/1103P and 1103P/1103P suggests that the PN scion particularly alters ethylene signalling and amino acid biosynthesis.

## Conclusion

Here we showed that short-term (27 h) of LP treatment in grafted grapevine resulted in the differential regulation of many genes in the roots; a core set of genes DE in all scion/rootstock in response to LP was identified, which surprisingly contained only few of the PSI genes identified in Arabidopsis (although four of these genes were induced after 28 d of treatment). This core set of DE genes contained many more down- than up-regulated genes and many genes related to the synthesis of stilbenes were present in the list of down-regulated genes. The comparison of the response to LP between the two homo-grafts highlighted the genotype specific variation in transcriptional response. Superimposed upon the genotype specific variation in transcriptional response to LP, the scion also modified the rootstock gene expression response to LP in a rootstock genotype specific fashion. Weighted gene co-expression network analysis identified genes clusters correlated with P supply, and the rootstock and scion genotype. This data set suggests that some potential phloem mobile signals associated with P supply were differentially regulated in the different scion/rootstocks genotypes studied; these signals include metabolites, cysteine rich peptides, hormones such as strigolactones and ethylene, and microRNAs. Although these signals are known to convey messages from the shoot to the root in model species, here we show that genetic variation in a crops species can potentially alter these signalling events in grafted plants. This study highlights the importance of studying grafted plants during rootstock selection and that the scion genotype can modify root responses to the environment.

## Methods

### Plant material and growing conditions

An American rootstock genotype, the *V. berlandieri* x *V. rupestris* hybrid cv. ‘1103 Paulsen’ (1103P, clone 198; Vitis international variety catalogue number: 9023), was collected from a vineyard in Bordeaux, France (according to institutional guidelines). The identification of 1103P was done by the Centre de Ressources Biologiques de la Vigne, collection ampélographique de Vassal, Montpellier, France, by simple sequence repeats markers. The *V. vinifera* genotype used was obtained from the sexual reproduction from self-pollination of Pinot Noir, named 40,024 (PN), and was provided by INRAE Colmar, France, and its genome was sequenced by a French-Italian consortium [62] and it also grows and grafts well in in vitro culture. No permissions were required to obtain this plant material. Both genotypes were introduced into in vitro culture after surface sterilisation. All four possible scion/rootstock combinations were micro-grafted in vitro using the cleft grafting system, i.e. 1103P/1103P, 1103P/PN, PN/1103P and PN/PN. Plants were cultivated in vitro on McCown Woody Plant Medium (Duchefa) supplemented with 30 g L^− 1^ sucrose, 0.27 μM 1-naphthalene acetic acid and 0.4% agar in a growth chamber at 22 °C and with a photoperiod of 16 h light/8 h dark at a photon flux density of 55 μmol m^− 2^ s^− 1^. Six-week-old plantlets were then acclimated for 4 weeks to perlite-filled pots, irrigated with water, in a growth chamber at 26 °C and with a photoperiod of 16 h light/8 h dark at a photon flux density of 145 μmol m^− 2^ s^− 1^ at plant level. Plants were then transferred into hydroponic culture with an aerated solution; each pot contained 2 plants of the same scion/rootstock combination with 700 mL of high P solution (HP, 0.6 mM P). Four days later, half the pots continued receiving the HP solution and the other half were subjected to a low P (LP, 0.001 mM P) treatment. The macronutrient composition was 2.45 mM KNO_3_, 0.69 mM MgSO_4_ and 1.27 mM CaCl_2_ for both the HP and LP solutions; HP solution also contained 0.6 mM KH_2_PO_4_ and 0.6 mM CaSO_4_, whereas the LP solution contained 0.3 mM K_2_SO_4_ and 0.3 mM CaSO_4_. Micronutrients were supplied as 46 μmol H_3_BO_3_, 9.1 μmol MnCl_2_, 2.4 μmol ZnSO_4_, 0.5 μmol CuSO_4_ and 14 nmol (NH_4_)_6_Mo_7_O_24_, and iron was supplied as 8.5 mg L^− 1^ Sequestrene 138 (i.e. 31.3 μmol ethylenediamine-N,N′-bis (2-hydroxyphenylacetic acid) NaFe, Syngenta Agro S.A.S.). The solutions were changed once a week for 28 d.

### RNA extraction

After 27 h and 28 d of LP or HP treatment, three pools of three root tips per plant (~ 15 mm in length) were harvested and immediately snap-frozen in liquid N for RNA sequencing (RNA-Seq) and quantitative reverse transcription polymerase chain reaction (RT-qPCR) analysis respectively. Total RNA of samples was extracted using the Spectrum Plant Total RNA kit (Sigma-Aldrich) with some modifications as previously described [54].

### RT-qPCR analysis

Total RNA (1.5 μg) was reverse transcribed into cDNA using the SuperScript III First-Strand Synthesis System (Invitrogen). Quantitative reverse transcription polymerase chain reactions were performed using SYBR Green on an iCycler iQH (Bio-Rad) according to the procedure described by the supplier, with 0.2 μM of primers for each gene. Gene expression was calculated as normalized relative quantities [63] with three reference genes. Primer sequences are listed in Additional File 12. Statistical analysis was done using the software R [64]. Data were analysed using a two-way analysis of variance (*p* < 0.05, with Tukey’s Honest Significant Difference test).

### RNA sequencing

RNA sequencing libraries were generated from 500 ng of total RNA using TruSeq Stranded mRNA LT Sample Preparation Kit (Illumina), according to manufacturer’s instructions. These libraries were then sequenced using Illumina Hiseq 4000 to produce 50 bp paired-end reads following Illumina’s instructions. The RNA-seq data has been deposited in the ArrayExpress database (http://www.ebi.ac.uk/arrayexpress [65]) under the accession number E-MTAB-8678.

### Pre-processing of RNA-Seq data

Adapter dimer reads were removed using DimerRemover (https://sourceforge.net/projects/dimerremover/) and quality of each sample was assessed with FastQC v0.11.2 [66]. Transcript abundance was quantified using salmon v0.13.1 [67] with the flags --seqBias --gcBias --fldMean 50 --fldSD 1 --validateMappings -rangeFactorizationBins 4. Quantification results were concatenated to a count matrix using tximport R package [68]. After quality verification, one sample was identified as outlier and discarded from subsequent analysis.

### Genome functional annotation

Gene models of the V3 annotation [69] were searched against the Araport11 (release 06.17.16, https://www.araport.org/) protein database with the blastx function of the DIAMOND version 0.9.19 software set to default parameters and reporting alignments in the 1% range of the top alignment score [70]. For each V3 gene model, the best blast hit was kept (1-to-1) and for multiple hits with the same score (1-to-many), the first hit was kept as representative keeping the other hits accessible. The corresponding gene annotations were obtained from the Araport11 gff file (release 06.22.16) and correspondences for V1 IDs were downloaded (https://urgi.versailles.inra.fr/Species/Vitis/Annotations).

### Weighted gene co-expression network analysis (WGCNA)

A co-expression gene network was constructed using the WGCNA software package (v1.63) in R [71, 72]. After having filtered the low-expressed genes, 24,190 genes were used. The power β was set at 11. The module eigengenes were used to evaluate the association between the 26 identified gene modules and the experimental variables. Then, a kME value (module eigengene-based connectivity) was calculated for each gene with every module. For each module eigengene, highly correlated genes were filtered with a correlation coefficient > 0.80 and a *p*-value < 0.01 and used to perform the enrichment analysis described below.

Differential expression analysis and MapMan BIN enrichment.

The R package DESeq2 [73] was used to detect differentially expressed (DE) genes using the following thresholds: False Discovery Rate (FDR) adjusted *p*-value < 0.01 and |log2 fold change (LFC)| > 1. MapMan BIN codes [74] were attributed to each gene using Mercator 4 (http://plabipd.de/portal/mercator-ii-alpha-version-). Enrichment analysis was performed using Fisher’s test, results with a Bonferroni’s adjusted *p*-value <= 0.05 and an enrichment > 1 were selected.

## Supplementary information

**Additional file 1.** Genes differentially expressed between high or low phosphate treatments (HP and LP respectively) in four scion/rootstock combinations of grapevine.

**Additional file 2.** Genes differentially expressed between the roots of two grapevine genotypes grown grafted under high or low phosphate supply (HP and LP respectively).

**Additional file 3.** MapMan BINs enriched in the genes differentially expressed in the roots between the two grapevine genotypes grown grafted under high phosphate conditions.

**Additional file 4.** Genes with a significant interaction in their gene expression response to low versus high phosphate supply between two homo-grafts of grapevine

**Additional file 5.** MapMan BINs enriched in the genes differentially expressed in the roots in response to low phosphate between two homo-grafts of grapevine: *Vitis vinifera* cv. Pinot noir and *V. rupestris x V. berlandieri* cv. 1103 Paulsen.

**Additional file 6.** Genes differentially expressed between hetero- and homo-grafts of grapevine grown under either high or low phosphate supply.

**Additional file 7.** MapMan BINs enriched in the 24 genes down-regulated in the roots between the scion/rootstock combinations 1103P/PN versus PN/PN grown under both high and low phosphate supply. PN: *Vitis vinifera* cv. Pinot noir; 1103P: *V. rupestris* x *V. berlandieri* cv. 1103 Paulsen.

**Additional file 8.** MapMan BINs enriched in the 661 and 505 genes up- and down-regulated respectively in the roots between the scion/rootstock combinations PN/1103P versus 1103P/1103P grown under both high and low phosphate supply. PN: *Vitis vinifera* cv. Pinot noir; 1103P: *V. rupestris* x *V. berlandieri* cv. 1103 Paulsen.

**Additional file 9.** Genes with a significant interaction in their gene expression response to low versus high phosphate supply between the scion/rootstock combinations 1103P/PN and PN/PN. PN: *Vitis vinifera* cv. Pinot noir; 1103P: *V. rupestris* x *V. berlandieri* cv. 1103 Paulsen.

**Additional file 10.** Genes with a significant interaction in their gene expression response to low versus high phosphate supply between the scion/rootstock combinations PN/1103P and 1103P/1103P. PN: *Vitis vinifera* cv. Pinot noir; 1103P: *V. rupestris* x *V. berlandieri* cv. 1103 Paulsen.

**Additional file 11.** Gene module membership. Module eigengene-based connectivity values for each gene in each module identified in a WGCNA analysis of genes expressed in the roots of grafted grapevine grown under two phosphate supplies.

**Additional file 12.** Primers used for RT-qPCR experiments.

## Data Availability

The datasets generated during the current study are available in the ArrayExpress database (http://www.ebi.ac.uk/arrayexpress [65]) under the accession number E-MTAB-8678.

## Abbreviations

1103P: *Vitis berlandieri* x *V. rupestris* cultivar 1103 Paulsen
4CL: 4-COUMAROYL COA-LIGASE;
ABA: Abscisic acid
bHLH: basic helix-loop-helix
C4H: CINNAMATE-4-HYDROXYLASE
DE: Differentially expressed
ERF: ETHYLENE RESPONSE FACTOR
HP: High phosphorus
IPS: INDUCED BY PHOSPHATE STARVATION1
LP: Low phosphorus
NLR: NUCLEOTIDE-BINDING DOMAIN AND LEUCINE-RICH REPEAT-CONTAINING
P: Phosphorus
PAL: PHENYLALANINE AMMONIA-LYASE
PHO2: PHOSPHATE2
PHR1: PHOSPHATE STARVATION RESPONSE1
PHT1: PHOSPHATE TRANSPORTER1
Pi: Orthophosphate
PN: *V. vinifera* cultivar Pinot Noir
PSI: Phosphate-starvation-inducible
RNA-Seq: RNA-sequencing
RT-qPCR: quantitative reverse transcription polymerase chain reaction
SPX: SYG/PHO81/XPR1
STS: STILBENE SYNTHASE
TF: Transcription factor
TKL: TYROSINE KINASE-LIKE
VIT: VACUOLAR IRON TRANSPORTER
WGCNA: weighted gene co-expression network analysis.
