# Supplementary material for: Scion genotypes exert long distance control over rootstock transcriptome responses to low phosphate in grafted grapevine
Source: BMC Plant Biol. 2020 Aug 3;20:367. doi: 10.1186/s12870-020-02578-y (PMC7398338; doi:10.1186/s12870-020-02578-y)
Supplement: Supplementary file 3 — Additional file 3. MapMan BINs enriched in the genes differentially expressed in the roots between the two grapevine genotypes grown grafted under high phosphate conditions. [file 12870_2020_2578_MOESM3_ESM.docx]

Additional File 3. MapMan BINs enriched in the genes differentially expressed in the roots between the two grapevine genotypes grown grafted under high phosphate conditions: enrichment of genes more highly expressed in *Vitis vinifera* cv. Pinot noir (PN) and *V. rupestris* x *V. berlandieri* cv. 1103 (1103P) (with both a PN or 1103P scion).

|  | **BIN** | **Name** | **Enrichment** | **Adjusted p-value** |
| --- | --- | --- | --- | --- |
| Genes more highly expressed in PN | 16.9.1.2.1 | RNA processing.messenger ribonucleoprotein particle (mRNP).mRNP export.TREX-2 mRNP trafficking complex.GANP/SAC3 scaffold component | 12.6 | 0.03 |
|  | 18.12.1.4 | Protein modification.S-glutathionylation and deglutathionylation.glutathione S-transferase activities.class tau | 4.9 | 0.00 |
|  | 26.6.2.1 | External stimuli response.biotic stress.pathogen effector.NLR effector receptor | 5.1 | 0.00 |
|  | 35.1 | not assigned.annotated | 1.2 | 0.04 |
|  | 50.1.10 | Enzyme classification.EC_1 oxidoreductases.EC_1.10 oxidoreductase acting on diphenol or related substance as donor | 3.2 | 0.03 |
|  | 50.1.13 | Enzyme classification.EC_1 oxidoreductases.EC_1.14 oxidoreductase acting on paired donor with incorporation or reduction of molecular oxygen | 3.0 | 0.00 |
|  | 50.2.4 | Enzyme classification.EC_2 transferases.EC_2.4 glycosyltransferase | 2.6 | 0.00 |
|  | 50.2.7 | Enzyme classification.EC_2 transferases.EC_2.7 transferase transferring phosphorus-containing group | 1.9 | 0.00 |
|  | 9.2.1.1 | Secondary metabolism.phenolics.p-coumaroyl-CoA synthesis.phenylalanine ammonia lyase (PAL) | 8.4 | 0.04 |
|  | 9.2.2.1 | Secondary metabolism.phenolics.flavonoid synthesis and modification.chalcone synthase | 6.8 | 0.00 |
| Genes more highly expressed in 1103P | 18.12.1.4 | Protein modification.S-glutathionylation and deglutathionylation.glutathione S-transferase activities.class tau | 3.7 | 0.01 |
|  | 26.6.2.1 | External stimuli response.biotic stress.pathogen effector.NLR effector receptor | 6.3 | 0.00 |
|  | 35.1 | not assigned.annotated | 1.4 | 0.00 |
|  | 50.1.10 | Enzyme classification.EC_1 oxidoreductases.EC_1.10 oxidoreductase acting on diphenol or related substance as donor | 3.1 | 0.01 |
|  | 50.1.13 | Enzyme classification.EC_1 oxidoreductases.EC_1.14 oxidoreductase acting on paired donor with incorporation or reduction of molecular oxygen | 3.2 | 0.00 |
|  | 50.1.3 | Enzyme classification.EC_1 oxidoreductases.EC_1.3 oxidoreductase acting on CH-CH group of donor | 4.6 | 0.00 |
|  | 50.2.1 | Enzyme classification.EC_2 transferases.EC_2.1 transferase transferring one-carbon group | 3.5 | 0.00 |
|  | 50.2.4 | Enzyme classification.EC_2 transferases.EC_2.4 glycosyltransferase | 3.3 | 0.00 |
|  | 50.3.2 | Enzyme classification.EC_3 hydrolases.EC_3.2 glycosylase | 2.5 | 0.00 |
