# Supplementary material for: Scion genotypes exert long distance control over rootstock transcriptome responses to low phosphate in grafted grapevine
Source: BMC Plant Biol. 2020 Aug 3;20:367. doi: 10.1186/s12870-020-02578-y (PMC7398338; doi:10.1186/s12870-020-02578-y)
Supplement: Supplementary file 5 — Additional file 5. MapMan BINs enriched in the genes differentially expressed in the roots in response to low phosphate between two homo-grafts of grapevine: Vitis vinifera cv. Pinot noir and V. rupestris x V. berlandieri cv. 1103 Paulsen. [file 12870_2020_2578_MOESM5_ESM.docx]

Additional File 5. MapMan BINs enriched in the genes differentially expressed in the roots in response to low phosphate between two homo-grafts of grapevine: *Vitis vinifera* cv. Pinot noir and *V. rupestris x V. berlandieri* cv. 1103 Paulsen.

| **BIN** | **Name** | **Enrichment** | **Adjusted p value** |
| --- | --- | --- | --- |
| 15.7.17 | RNA biosynthesis.transcriptional activation.NAC transcription factor | 10.6 | 0.00 |
| 15.7.22 | RNA biosynthesis.transcriptional activation.WRKY transcription factor | 11.1 | 0.01 |
| 24.2.12.3 | Solute transport.carrier-mediated transport.VIT family.iron cation transporter (VTL-type) | 53.7 | 0.04 |
| 24.3.11 | Solute transport.channels.QUAC/ALMT anion channel | 28.9 | 0.01 |
| 26.6.2.1 | External stimuli response.biotic stress.pathogen effector.NLR effector receptor | 8.9 | 0.00 |
| 26.6.2.2.3 | External stimuli response.biotic stress.pathogen effector.ETI (effector-triggered immunity) network.EDS1 regulator | 53.7 | 0.04 |
| 50.1.10 | Enzyme classification.EC_1 oxidoreductases.EC_1.10 oxidoreductase acting on diphenol or related substance as donor | 8.7 | 0.00 |
| 50.2.7 | Enzyme classification.EC_2 transferases.EC_2.7 transferase transferring phosphorus-containing group | 2.9 | 0.00 |
