# Supplementary material for: Scion genotypes exert long distance control over rootstock transcriptome responses to low phosphate in grafted grapevine
Source: BMC Plant Biol. 2020 Aug 3;20:367. doi: 10.1186/s12870-020-02578-y (PMC7398338; doi:10.1186/s12870-020-02578-y)
Supplement: Supplementary file 7 — Additional file 7. MapMan BINs enriched in the 24 genes down-regulated in the roots between the scion/rootstock combinations 1103P/PN versus PN/PN grown under both high and low phosphate supply. PN: Vitis vinifera cv. Pinot noir; 1103P: V. rupestris x V. berlandieri cv. 1103 Paulsen. [file 12870_2020_2578_MOESM7_ESM.docx]

Additional File 7. MapMan BINs enriched in the 24 genes down-regulated in the roots between the scion/rootstock combinations 1103P/PN and PN/PN grown under both high and low phosphate supply. PN: *Vitis vinifera* cv. Pinot noir; 1103P: *V. rupestris* x *V. berlandieri* cv. 1103 Paulsen.

| \| **BIN** \| **Name** \| **Enrichment** \| **Adjusted p-value** \| \| --- \| --- \| --- \| --- \| \| 13.3.6.6.1 \| Cell cycle.mitosis and meiosis.meiotic recombination.meiotic exit.MS5/TDM1 meiotic exit regulator \| 268.6 \| 0.05 \| \| 18.1.5.4 \| Protein modification.N-linked glycosylation.oligosaccharyl transferase (OST) complex.OST4 component \| 940.3 \| 0.01 \| \| 18.12.1.4 \| Protein modification.S-glutathionylation and deglutathionylation.glutathione S-transferase activities.class tau \| 51.1 \| 0.01 \| \| 25.4.2.1 \| Nutrient uptake.iron uptake.reduction-based strategy uptake.Fe(III)-chelate reductase \| 470.1 \| 0.03 \| \| 25.5.2.1 \| Nutrient uptake.copper uptake.reduction-based uptake.FRO metal ion-chelate reductase \| 470.1 \| 0.03 \| \| 50.1.13 \| Enzyme classification.EC_1 oxidoreductases.EC_1.14 oxidoreductase acting on paired donor with incorporation or reduction of molecular oxygen \| 25.2 \| 0.00 \| |  |  |  |  |  |  |
| --- | --- | --- | --- | --- | --- | --- | --- | --- | --- | --- | --- | --- | --- | --- | --- | --- | --- | --- | --- | --- | --- | --- | --- | --- | --- | --- | --- | --- | --- | --- | --- | --- | --- | --- |
|  |  | | | |  |  |
|  |  | | | |  |  |
|  |  | | | |  |  |
|  |  | | | |  |  |
|  |  | | | |  |  |
|  |  | | | |  |  |
