# Supplementary material for: Scion genotypes exert long distance control over rootstock transcriptome responses to low phosphate in grafted grapevine
Source: BMC Plant Biol. 2020 Aug 3;20:367. doi: 10.1186/s12870-020-02578-y (PMC7398338; doi:10.1186/s12870-020-02578-y)
Supplement: Supplementary file 8 — Additional file 8. MapMan BINs enriched in the 661 and 505 genes up- and down-regulated respectively in the roots between the scion/rootstock combinations PN/1103P versus 1103P/1103P grown under both high and low phosphate supply. PN: Vitis vinifera cv. Pinot noir; 1103P: V. rupestris x V. berlandieri cv. 1103 Paulsen. [file 12870_2020_2578_MOESM8_ESM.docx]

Additional File 8. MapMan BINs enriched in the 661 and 505 genes up- and down-regulated respectively in the roots between the scion/rootstock combinations PN/1103P and 1103P/1103P grown under both high and low phosphate supply. PN: *Vitis vinifera* cv. Pinot noir; 1103P: *V. rupestris* x *V. berlandieri* cv. 1103 Paulsen.

|  | **BIN** | **Name** | **Enrichment** | **Adjusted p-value** |
| --- | --- | --- | --- | --- |
| Up-regulated | 15.7.1.5 | RNA biosynthesis.transcriptional activation.C2C2 superfamily.DOF transcription factor | 17.9 | 0.00 |
|  | 15.7.17 | RNA biosynthesis.transcriptional activation.NAC transcription factor | 7.8 | 0.00 |
|  | 15.7.3.5 | RNA biosynthesis.transcriptional activation.HB (Homeobox) superfamily.BEL transcription factor | 24.5 | 0.00 |
|  | 15.7.33 | RNA biosynthesis.transcriptional activation.bHLH transcription factor | 4.7 | 0.01 |
|  | 18.8.1.19 | Protein modification.phosphorylation.TKL kinase superfamily.L-lectin kinase | 9.3 | 0.01 |
|  | 19.5.2.1.1 | Protein degradation.peptidase families.serine-type peptidase activities.subtilisin-type protease families.SBT1 protease | 14.7 | 0.00 |
|  | 21.1.1.1.2.2 | Cell wall.cellulose.synthesis.cellulose synthase complex (CSC).CSC-interacting proteins.TED-type CSC-interactive protein | 50.9 | 0.00 |
|  | 26.3.2.5.1 | External stimuli response.temperature.Hsp (heat-shock-responsive protein) families.sHsp (small heat-shock-responsive protein) families.class-C-I protein | 13.4 | 0.03 |
|  | 3.4.1 | Carbohydrate metabolism.raffinose family oligosaccharide biosynthesis.galactinol synthase | 29.0 | 0.00 |
|  | 50.1.12 | Enzyme classification.EC_1 oxidoreductases.EC_1.13 oxidoreductase acting on single donor with incorporation of molecular oxygen (oxygenase) | 17.0 | 0.01 |
|  | 50.1.13 | Enzyme classification.EC_1 oxidoreductases.EC_1.14 oxidoreductase acting on paired donor with incorporation or reduction of molecular oxygen | 5.6 | 0.00 |
| Down-regulated | 11.10.2.1.1 | Phytohormones.signalling peptides.CRP (cysteine-rich-peptide) category.GASA/GAST family.GASA precursor polypeptide | 18.5 | 0.01 |
|  | 15.7.1.3 | RNA biosynthesis.transcriptional activation.C2C2 superfamily.GATA transcription factor | 24.9 | 0.03 |
|  | 15.7.33 | RNA biosynthesis.transcriptional activation.bHLH transcription factor | 4.9 | 0.03 |
|  | 15.7.35.1 | RNA biosynthesis.transcriptional activation.GRF-GIF transcriptional complex.GRF transcription factor component | 50.1 | 0.00 |
|  | 19.5.2.2 | Protein degradation.peptidase families.serine-type peptidase activities.serine carboxypeptidase | 12.4 | 0.00 |
|  | 50.1.10 | Enzyme classification.EC_1 oxidoreductases.EC_1.10 oxidoreductase acting on diphenol or related substance as donor | 6.1 | 0.01 |
|  | 50.3.2 | Enzyme classification.EC_3 hydrolases.EC_3.2 glycosylase | 4.8 | 0.00 |
