# Supplementary material for: Scion genotypes exert long distance control over rootstock transcriptome responses to low phosphate in grafted grapevine
Source: BMC Plant Biol. 2020 Aug 3;20:367. doi: 10.1186/s12870-020-02578-y (PMC7398338; doi:10.1186/s12870-020-02578-y)
Supplement: Supplementary file 12 — Additional file 12. Primers used for RT-qPCR experiments. [file 12870_2020_2578_MOESM12_ESM.docx]

Additional File 12. Primers used for RT-qPCR experiments.

| **Gene name** | **Accession number** | **Forward primer** | **Reverse primer** | **Efficiency** |
| --- | --- | --- | --- | --- |
| SPX1/SPX2 | Vitvi11g00466 | GATGGACAGGAAGGGTGTGG | TTTCCTTCAGAGCCCGCAAT | 91 |
| SPX3 | Vitvi05g00882/ Vitvi15g00674 | CAGGAACACAGTTGCAGCAC | TGGGGATGGGAGAGTGGAAT | 91.5 |
| PHT 1;4b | Vitvi05g00734 | CCCACCTGGTATTGGAATGAGA | TAGTAGGAGGCTGCATGTCCA | 85 |
| PHT 1;4c | Vitvi05g01956 | CCCGAGTCCAAGGGAAAGTC | GGAACAGTCCTAGCCTGCTG | 99.1 |
| ACTIN | Vitvi02g00281/ Vitvi04g01613 | CTTGCATCCCTCAGCACCTT | TCCTGTGGACAATGGATGGA | 93.6 |
| GAPDH | Vitvi17g01598 | CCACAGACTTCATCGGTGACA | TTCTCGTTGAGGGCTATTCCA | 91 |
| SAND3' | Vitvi06g00278 | TGCTGGGTTACCCCGGAGTTTGA | CAGACCCGGTTGCACGTCCG | 89.9 |
